# How well do the adult social care outcomes toolkit for carers, carer experience scale and care-related quality of life capture aspects of quality of life important to informal carers in Australia?

**DOI:** 10.1007/s11136-023-03459-1

**Published:** 2023-06-25

**Authors:** Jessica Bucholc, Nikki McCaffrey, Anna Ugalde, Anne Muldowney, Stacey Rand, Renske Hoefman, Cathrine Mihalopoulos, Lidia Engel

**Affiliations:** 1 Deakin Health Economics, Institute for Health Transformation, School of Health and Social Development, Deakin University, Geelong, VIC Australia; 2 Quality and Patient Safety, Institute for Health Transformation, School of Nursing and Midwifery, Deakin University, Geelong, VIC Australia; 3 Older Persons Advocacy Network, Sydney, Australia; 4 Personal Social Services Research Unit (PSSRU), Cornwallis Building, University of Kent, Canterbury, UK; 5 The Netherlands Institute for Social Research (SCP), The Hague, The Netherlands; 6 Health Economics Division, School of Public Health and Preventive Medicine, Monash University, Melbourne, Melbourne, VIC Australia

**Keywords:** Informal care, Outcome measurement, Carer-related quality of life, Preference-based measures

## Abstract

**Purpose:**

Identify aspects of quality of life (QoL) important to Australian informal carers and explore how well the Adult Social Care Outcomes Toolkit for Carers, Care-related Quality of Life instrument and Carer Experience Scale capture these aspects in the Australian context.

**Methods:**

Online questionnaires were completed by Australian informal carers. Socio-demographics, open-ended questions: positive/negative aspects of caring and QoL aspects missing from the instruments, and ranking of the instrument domains was used to explore the content of the instruments. Instruments were scored using preference-weighted value sets (reported in another paper). Content analysis was used to analyse the open-ended responses. Chi-squared test looked at differences in domain importance. Descriptive analyses summarised all other information.

**Results:**

Eight themes were identified: Behaviour-mood of the care recipient, Caring responsibilities, Finances, Health, Own life, Perception of carers, Relationship with care recipient and Support. Many aspects of carer QoL mentioned as missing in the instruments appeared covered by the domains, of which all were reported as important. The highest ranked domain was relationship with the care recipient. The influence of the care recipient specific support, behaviour/mood and health on carer QoL appear absent in all instruments.

**Conclusion:**

The content of the three instruments appears relevant in an Australian setting. The influence of care recipient’s health and well-being on carer QoL should be considered, along with spillover effects. A content and/or face validity analysis is required to confirm differences in item interpretation in Australian informal carers.

**Supplementary Information:**

The online version contains supplementary material available at 10.1007/s11136-023-03459-1.

## Background

Internationally, there is a growing call to include informal carer costs and benefits in economic evaluations to evaluate the broader impacts of health and social care services [1, 2]. In this context, informal carers provide care beyond normal expectations within a pre-existing relationship (family member, relation, friend, neighbour) such as assistance with personal care, household activities or practical support, and generally do not receive payment for the care they provide [3, 4]. Over the last 15 years, three carer-specific preference-based instruments have been developed to measure outcomes for economic evaluations, the Adult Social Care Outcomes Toolkit for Carers (ASCOT-Carer) [5], Care-related Quality of Life instrument (CarerQol) [6] and Carer Experience Scale (CES) [7]. Whilst the number of economic evaluations including carer effects has grown in recent years, few have been conducted in the Australian setting [2, 8-10].

In Australia, there are about 2.8 million informal carers with over a third acting as the primary carer, i.e. the person who provides the majority of care [11]. In 2020, informal carers provided an estimated 2.2 billion hours of care, on average 786 h per year or 15 h per week (35.2 h for primary carers) [11, 12]. If services were purchased from formal care providers, the replacement costs would be $77.9 billion, almost 40% of the total spending on health in Australia in the same year [11, 13]. Given societies implicit reliance on carers’ willingness to fulfil this role and the economic consequences if this situation should adversely change, it is crucial that carer costs and effects are considered in economic evaluations [11]. In turn, this would also better inform healthcare decision-makers on actual societal costs, increasing the chance that welfare optimising decisions are made.

Two recent studies have investigated the relative construct and discriminative validity, test–retest reliability and responsiveness of the European-developed ASCOT-Carer, CarerQol and CES in a survey of Australian carers [14, 15]. Studies in England have also compared ASCOT-Carer, CES, CarerQol and EuroQol-5 Dimension-5 level (EQ5D-5L) [14, 16]. These studies indicate that the instruments tap into different constructs of carer-related QoL and caring experiences, reflecting the original purpose of the instruments and suggests the ASCOT-Carer, CES and CarerQol cannot be used interchangeably [16, 17]. The ASCOT-Carer was developed to measure social care-related QoL and support of carers in the setting of policy and formal support interventions [5, 18]. Whereas the CarerQol measures the impact of informal care on carers’ QoL, combining the burden of caring and valuation of their well-being (happiness) in the context of an evaluation in health care [6]. The CES captures the caring experience rather than carer’s QoL per se [19]. Validation has been investigated for the constructs in each of the instruments: the ASCOT-Carer with carers in England [5]; the CES with carers of older people in England [19] and the CarerQol with carers in eight European countries [6] [20]. Each of the instruments has preference weights, allowing the calculation of a summary score which reflects carers’ preferences for difference aspects of carer-related QoL [7, 21-23].

An Australian population was only included in one of the CarerQoL validation studies [22], with the general adult population using hypothetical carer scenarios. Content validation of instruments assesses relevance, comprehensiveness, and comprehensibility of the questions and the overall instrument; ensuring interpretation is as intended, all aspects important to the specific population are included and that the instrument’s constructs/domains measured as proposed [24]. Cross-cultural adaptations of instruments are important to capture differences in linguistics, colloquialisms, context and culture, even if translation is not required [25]. Qualitative research is ideally placed to validate the face value and content of instruments by exploring these social and cultural variables that may differ between informal carers in different countries [26]. Given the differences in health and social care support between countries [27-29], it is important to evaluate the applicability of these instruments in an Australian setting [25].

Consequently, the aims of this analysis were to identify aspects of carer QoL important to Australian informal carers and explore how well the ASCOT-Carer, CarerQol and CES constructs capture these aspects in the Australian context.

## Methods

### Study design

An online questionnaire was administered to a sample of informal carers in Australia between June and September 2018. Participants were recruited through Carers Victoria, a state-wide not-for-profit organisation supporting carers to improve their wellbeing, health, resilience and capacity [30]. This analysis was part of a larger study that investigated the psychometric properties of the carer-related preference-based instruments [15] and exploratory factor analysis [17].

### Setting and participants

Adults (≥ 18 years), Australian residents who self-identified as primary, informal carers and able to read the English written study questionnaire were invited to complete a web-based questionnaire. An email invitation was sent to all Carers Victoria registered informal carers who had previously consented to contact for research purposes. The online questionnaire link was also advertised in the *Voice: Carers Victoria ebulletin* which is distributed to all informal carers registered with the organisation and in researcher’s social media posts. Informed consent was collected before starting the questionnaire and a $10 gift voucher was offered to all participants as an acknowledgement of their contributions.

### Instruments

#### Adult Social Care Outcomes Toolkit for Carers (ASCOT-Carer)

There are seven domains in the ASCOT-Carer, a preference-based instrument of carers’ social care-related quality of life including; control over daily life, occupation (doing things you value and enjoy), social participation and involvement, personal safety, self-care, time and space to be yourself and feeling supported and encouraged [5, 21]. The content of the ASCOT-Carer was developed from a literature review, focus groups and interviews with carers and care managers [31], and semi-structured interviews with carers [18, 32].

#### Care-related Quality of Life (CarerQol)

CarerQol contains two sections; the CarerQol-Visual Analogue Scale (VAS), which measures wellbeing and the CarerQol-7D which measures subjective burden [6, 33]. There are seven dimensions in the latter; fulfilment, support, relational problems, mental health problems, problems combining daily activities with care, financial problems and physical health problems. The content of the CarerQol was developed from a survey of carers in the Netherlands and a review of eight popular burden measures [6, 23].

#### Carer Experience Scale (CES)

There are six dimensions in the CES, a preference-based instrument of caring experiences; activities outside caring, support from family and friends (social support), assistance from organizations and the government (institutional support), fulfilment from caring, control over the caring and getting on with the care recipient [7]. The content of the CES was developed from semi-structured interviews with carers in the UK and a meta-ethnography of qualitative studies on caring.

### Questionnaire

The questionnaire was developed online using Qualtrics®. It was piloted to refine wording and comprehension of the activities with a convenience sample of Deakin University Health Economics and Faculty of Health staff members and informal carers (n = 21).

**Fig. 1:**
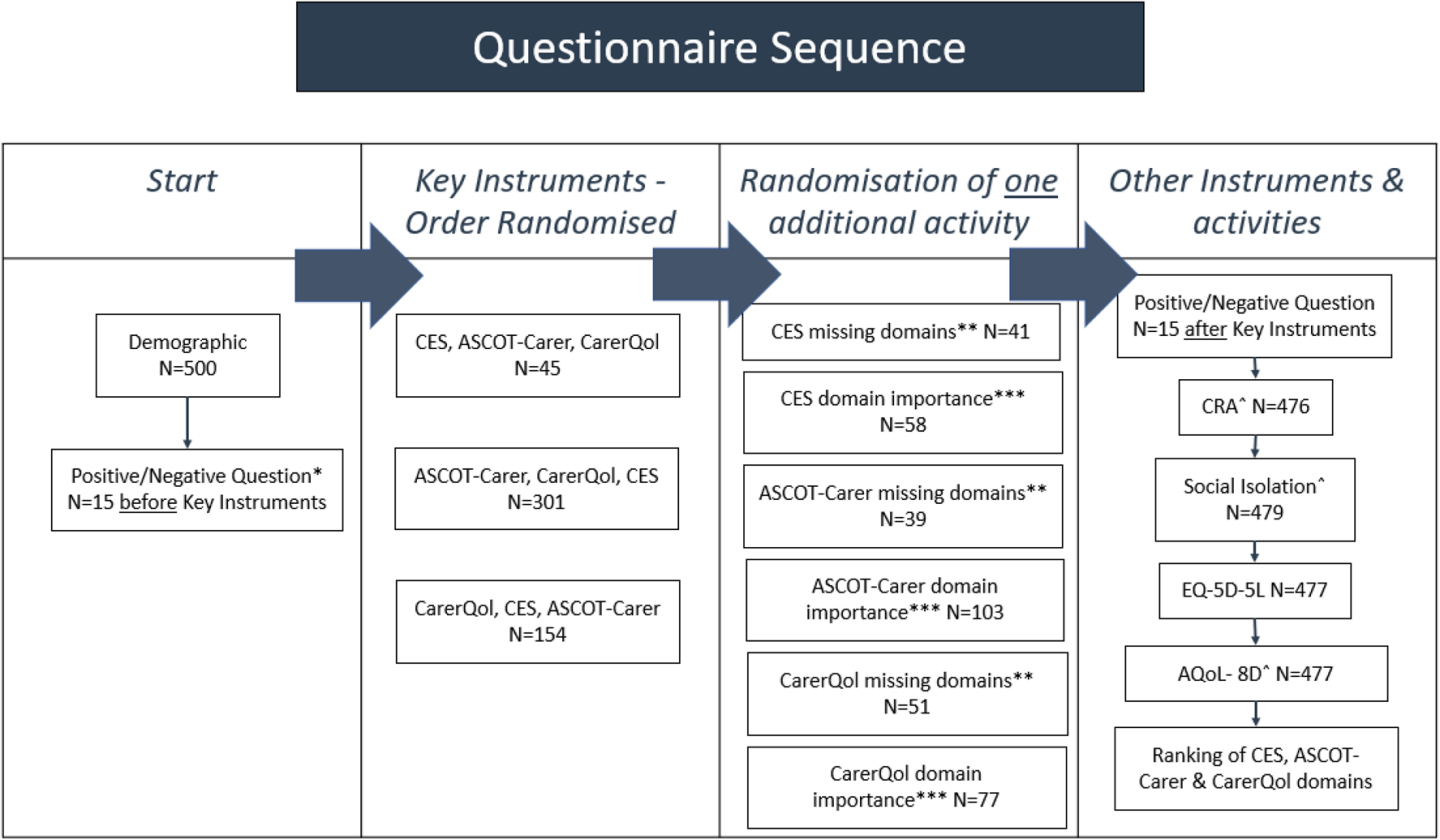
Questionnaire Flow. *Positive/Negative questions: Please describe the things that have had the greatest positive effect on your quality of life as a carer in the past week?; Please describe the things that have had the greatest negative effect on your quality of life as a carer in the past week? (Response free text). ** [Instrument] followed by question: Thinking about the statements included in this completed questionnaire, please describe any other things that affect your quality of life as a carer that were NOT mentioned. (response free text). *** [Instrument] and following each question: How important is this to your quality of life as a carer? (Response 5-point Likert scale, Very Important to Not Important at all). ˆCRA = Caregiver Reaction Assessment; Social Isolation = Three item UCLA Loneliness Scale; AQoL-8D = Assessment of Quality of Life- 8 Dimensions

Study participants had the option to complete the questionnaire over multiple sessions and all questions were voluntary. Figure 1 shows the sequence of instruments, randomisations and the question wording.

Firstly, contextual questions about personal characteristics, caring situation and characteristics of the care recipient were asked. Followed by the randomised ASCOT-Carer, CarerQol and CES instruments to minimize potential ordering effects [15]. To achieve the aims of this paper, the first thirty participants were asked two separate free text questions about the greatest positive and greatest negative effect on their quality of life as a carer in the past week.

To minimise survey burden, all participants were randomised to one additional activity: (i) instrument plus free text question on whether any aspects of carer QoL were not mentioned in the instrument (referred to in this paper as ‘[Instrument] missing domains’); (ii) instrument plus a 5-point Likert scale of the importance of each domain within the instrument s (referred to in this paper as ‘[Instrument] domain importance’); or (iii) complete the instrument only.

Finally, all respondents were invited to rank the top five (out of the 14 total domains across the three instruments) most important aspects of caring from most to least relevant (referred to in this paper as ‘ranking activity’).

Other instruments were also completed by all participants in the final part of the questionnaire (Fig. 1, last column), for use in the broader project [15, 17].

### Data analysis

Analyses were conducted in STATA Statistical Software: Release 17 [34], QSR NVivo software© (version 11) [35] and Microsoft Excel [36]. Instruments were scored using preference-based weighting of the respective instruments. To ensure consistency, the UK value sets were used for all three instruments. Results were reported in a previous paper [15].

### Population

Descriptive statistics were generated for the demographics, caring situation and care recipient characteristics.

### Relevance and comprehensiveness

#### Relevance

Responses to the Positive/Negative questions and [Instrument] missing domains questions, were imported into QSR NVivo software version 11 [35] for analysis to identify specific factors influencing respondents’ care-related QoL. A four-stage content analysis procedure guided the coding of the open-ended responses: decontextualization, recontextualization, categorisation, and compilation [37]. Conventional content analysis was used in the development of the coding framework and themes were inductive, data driven, and with researchers avoiding using preconceived categories [37, 38].

Responses for each instrument and question were coded separately. Coder one (JB) spent time noting any preliminary ideas, codes and themes before building categories and with these, a coding structure. Where responses contained more than one theme/sub-theme they were coded into each.

Coder two (AU) reviewed the coding structure and relevant-free text responses. The two coders discussed differing views on codes and discrepancies were settled by authors LE and NM before finalising the coding structure.

Proportions of the [Instrument] domain importance question rated as unimportant, neutral or important to respondents’ CrQoL were calculated and compared with the Chi-Square test.

In addition, for the ranking activity, descriptive analyses were used to determine number of times domains were ranked number one and also the number of times chosen in the top five ranks.

#### Comprehensiveness

The coding structure of the analysis for questions [Instrument] missing domains was reviewed by authors JB & LE to identify any key aspects of carer QoL that participants identified as missing from the instruments.

## Results

### Population

Online Appendix 1 shows the sociodemographic characteristics and caring situation of the informal carers and care recipient characteristics of the total questionnaire sample and for each of the subgroups that received and completed the additional questions that contributed to this analysis.

The total sample size was 500 participants with a mean age of 52 and the mean age of the care recipient was 45. Majority were female (79%) and had completed undergraduate and postgraduate education (46%). Just over half of the participants were employed (51%), while the other half were retired or engaging in housework duties including caring (46%). Just over half of participants were sole carers and a quarter providing care to multiple recipients. Most participants shared a household with the care recipient (81%) and had been caring for > 24 months (74%). Relationships with care recipients included children (32%), parents (32%), partners (25%) and other family members or friends (10%) with their most common medical condition being chronic diseases or disabilities (44%) followed by mental health problems (33%).

### Relevance

The open-ended responses (N = 115) resulted in 244 units of data and identified eight themes: Behaviour-Mood of care recipient; Caring Responsibilities; Finances; Health; Own Life; Perceptions of carers; Relationship with care recipient; and Support. Although the qualitative positive/negative and Instrument [missing domain] question responses were coded separately, strong similarities between the coding frameworks were very apparent with the same themes present for each question, differences only in sub-themes. Each question appeared to elicit responses that indicated what matters to carers, so the results have been presented together in Table 1 which summarises the themes and sub-themes presented in Online Appendix 2. There was a wide diversity in the open-ended responses. The number of responses ranged N = 24–33 for each open-ended question (the two Positive/Negative questions and three [Instrument] missing domain questions).

**Table 1:** Summary of ﻿Themes

Theme	Includes	Examples elicited from the Positive/Negative questions	Examples elicited from the [Instrument] missing domain questions
Behaviour-mood of care recipient	Actions and moods, physical and psychosocial actions of care recipient (e.g. abuse toward carer, unpredictability, happiness) that affect carer quality of life (QoL)	‘Seeing my son smile’ (Carer_242)	‘It is very hard to organise anything much as I never know what will unfold from one hour to another’ (Carer_679, ASCOT-Carer)
		‘My partner has been fairly cooperative and easy to get along with’ (Carer_5)	‘He gets upset and abusive and I start second guessing my decision’ (Carer_10, CarerQol)
		‘…she is highly suspicious about other members of the family so that I cannot enjoy my relationships with my son and grandchildren without having her having a reaction’ (Carer_11)	‘[Care recipient] put his hands on me until 15 years of age. It is abuse. I am very tired and feel like I will never fully recover’ (Carer_805, CES)
Caring responsibilities	Duties/tasks required of carers (e.g. advocating, travel required for caring). Also includes reason for caring including enjoyment and fulfilment of role	‘Having to again follow up on things that are in place and should be happening automatically, e.g. Webster Pac medications from pharmacy and form filling by Medical persons, but either are not ready when they should be or have been forgotten.’ (Carer_8)	‘The amount of time-consuming paperwork involved in caring—e.g. NDIA’ (Carer_120, CES)
			‘Long distances travelled to services’ (Carer_161, CarerQol)
			‘Even though the constant pressure is there, I do feel grateful that I am able to love and provide care and support for this wonderful person.’ (Carer_209, ASCOT-Carer)
Finances*	Costs of caring, income from employment, being able to work	‘Managed to go out for one coffee with a friend, there is not much time to do this regularly & I felt guilty for spending the money because finances are so tight & there are many medical appointments soon, & because if 2-h travel to services there’s very little time of finances to do the little things for me.’ (Carer_237)	‘the fact that I receive little financial support from government sources even though I am unable to work mainly because of my caring duties’ (Carer_245, CarerQol)
			‘Financial difficulties as I am only able to work part time as a [occupation], I cannot work during the day due to my having to provide support to my mum’ (Carer_705, *ASCOT-Carer)*
		‘Being late for work or having to leave early.” (Carer_241)	*‘Being able to balance caring role with work or study and life’* (Carer_279, CES)
Health	Physical, mental, and spiritual health and fatigue, relating to the carer or the care recipient (where it affects carer QoL)	‘Stress trying to complete all tasks necessary in limited time and not always in the right frame of mind. I'm often exhausted.’ (Carer_251)	‘I feel that there is a strong relationship with mum’s wellbeing and physical capacity and my own. I fear her deteriorating health condition will get out of my control, or her capacity to improve. If she deteriorates, I cannot have such a special time with her or take her places.’ (Carer_26, CarerQol)
			‘Don't sleep well as too much worry and thinking about the people I care for and what else I can do.’ (Carer_694, ASCOT-Carer)
		‘my current illness affecting my ability to be effective as a carer but also fear of passing my illness to my husband with the risk of serious complications for him. Minor illnesses can cause life threatening complications for him, so this is a constant fear for me’ (Carer_244)	‘My own disability and mental health issues, my husband’s disability and mental health issues’ (Carer_802, CES)
Own life	The impact of caring on personal life, socialising, family, exercise, control over own life due to caring	‘not feeling like I have the freedom to do the things that I would like to do’ (Carer_240)	‘Lack of time to see friends, enjoy, have fun, have weekends, have holidays’ (Carer_30, CarerQol)
		‘My faith, catching up with good friends and family, walking, enjoying time with my husband and son.’ (Carer_16)	Just being too tired to follow my own interests and if making plans to go out solo’ (Carer_24, CES)
		‘When my son is at school, I am able to exercise to ease stress’ (Carer_249)	‘my partner does not understand that he is very different from his stroke, mentally it’s hard for me to explain to him that l need time away from him’ (Carer_94, ASCOT-Carer
Perceptions of carers	Societal, community and family expectations of carers, including stigma of the caring role	‘Always the threat of argument and verbal abuse regarding the opinion that in today's society there is a lack of respect and children do not take responsibility for their elderly parents by looking after them in our own homes instead of 'abandoning them' in aged care facilities’ (Carer_17)	‘The negative and judgemental opinions of most members of my local ethnic migrant community about my not keeping my mother at my home with me instead of in a (well-functioning) nursing home even though she has dementia, is elderly, and a difficult person to live with…’ (Carer_149, CarerQol)
			‘Other external influences such as people's opinions, demands, expectations.’ (Carer_241, CarerQol)
			‘Guilt trips from people who value judge how we live’ (Carer_129, ASCOT-Carer)
Relationship with care recipient	Relationship between carer and care recipient including how it has changed, communication, feelings toward the care recipients and how time is spent together	‘He has been well enough, so we have been able to do more normal things together rather than just being a carer.’ (Carer _244)	‘You are no longer ' the person you used to be'. You no longer have the partner you once had. There is an incredible feeling of loss.’ (Carer_209, ASCOT-Carer)
			‘Disconnect with a relative suffering with dementia’ (Carer_17, CarerQol)
		‘Constantly having to think for another human being and listen to repetition after repetition. No ability to have any discussions’ Carer_3)	*‘Communication with him is very difficult’* (Carer_158, CES)
Support	Formal and informal support for carer and for care recipient. Includes government financial assistance	‘Scrambling to find a suitable Support worker for my son so that I can attend a meeting’ (Carer_10)	‘There is no support for carers that I know of. In the early days after my husband’s stroke there was some support, but everything is capped at a certain amount of visits. And after that you don’t meet the criteria anymore so you’re on your own. I have not had a proper break from my caring duties for more than 2 years. I care for my husband 24/7. No one works those hours in paid employment. I love my husband and wouldn’t have it any other way but to have support and know help is only a phone call away would be great.’ (Carer_108, ASCOT-Carer)
			‘When we look like caregivers who look like they have all the bases covered, you receive less support’ (Carer_778, CarerQol)
		‘this program has now been defunded so I won’t have the opportunity to meet with the other carers monthly/bimonthly and the support worker has lost his job so I will no longer have him as a support/source of assistance with my caring responsibilities’ (Carer_2)	‘Frustration with Government Departments and Caring Organisations who because they are underfunded, over committed or don't/can't do their jobs effectively. I am constantly told they can or will help then simply do not carry out their promises and or don't follow up as promised’ (Carer_179, CES)

*Government financial assistance coded to ‘Support’ theme

Likert responses to the importance of each domain were categorised into: Not Important (Not Important; Slightly Important), Neutral and Important (Important; Very Important) and aggregated within each instrument. The total instrument importance (Table 2) shows that carers in our sample judged all three instruments as important (≥ 80%) and ≤ 7% not important. There was no statistically significant difference between the ratings across the three instruments (Chi-squared 3.489, degrees of freedom 4, p = 0.479).

**Table 2:** Rating of importance of carer-related quality of life domains by Instruments

	Not Important n (%)	Neutral n (%)	Important n (%)	Total n
ASCOT-Carer	50 (7)	86 (12)	583 (81)	719
CarerQol	35 (7)	67 (13)	397 (80)	499
CES	17 (5)	38 (11)	293 (84)	348
Total	102 (7)	191 (12)	1273 (81)	1566

**Fig. 2:**
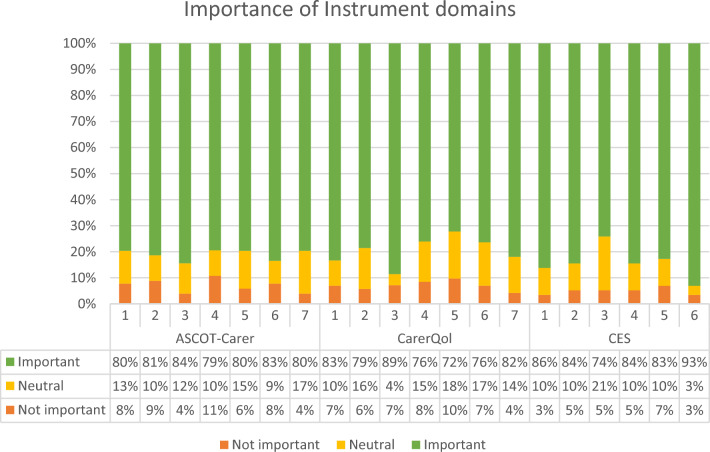
Importance of Instrument domains. *ASCOT-Carer Domains 1 = Occupation, 2 = Control over Daily Life, 3 = Self Care, 4 = Personal Safety, 5 = Social participation, 6 = Time and Space to be yourself, 7 = Feeling supported and encouraged. ** CarerQol Domains 1 = Fulfilment, 2 = Relational Problems, 3 = Mental Health Problems, 4 = Problems combining daily activities with care, 5 = Financial problems, 6 = Support, 7 = Physical Health problems. ***Carer Experience Scale Domains 1 = Activities outside Caring, 2 = Support from family and friends, 3 = Assistance from Organisations and Government, 4 = Fulfilment from Caring, 5 = Control over Caring, 6 = Getting on with the Care recipient

Similarly, the importance of individual domains within each instrument indicated that all the domains for each instrument were important aspects of CrQoL (Fig. 2). Domains most frequently considered important were the CES domain of getting on with the care recipient (n = 54, 93%) and activities outside of caring (n = 50, 86%) and CarerQol’s Mental Health Problems (n = 62, 89%).

Domains deemed least important were for ASCOT Carer’s personal safety (n = 11, 11%), and control over daily life (n = 9, 9%) and CarerQol’s financial problems (n = 7, 10%).

Table 3 summarises the instrument domain ranking activity. The top five ranked domains were the same using either analysis method (number of times ranked as position one or number of times ranked in the top 5), only the third and fourth positions (mental health and self-care) were reversed.

**Table 3:** Ranking of importance Activity- Number of times domains chosen as number one (most important) and number of times chosen in top five

Instrument (item)	CarerQol (2)** CES (6)***	CarerQol (7)**	CarerQol (3)**	ASCOT-Carer (3)*	ASCOT-Carer (6)*	CES (3)*** ASCOT-Carer (7)*	ASCOT-Carer (4)*
Rank/Domain	Relationship with care receiver	Physical health	Mental health	Self Care	Space and time to self	Institutional support	Safety
**1**	**98**	**48**	**42**	**45**	**24**	**27**	**22**
2	26	41	67	45	37	23	22
3	40	49	39	25	33	30	22
4	26	38	28	35	46	28	15
5	26	37	25	35	27	23	24
**Total**	**216**	**213**	**201**	**185**	**167**	**131**	**105**

Instrument (item)	ASCOT-Carer (1)* CarerQol (4)** CES (1)***	CarerQol (1)** CES (4)***	CareQol (5)**	ASCOT-Carer (7)* CarerQol (6)** CES (2)***	CES (5)***	ASCOT-Carer (5)*	ASCOT-Carer (2)*
Rank/Domain	Activities outside of caring	Fulfilment	Finance	Social Support	Control over caring	Social Participation	Control over daily life
**1**	**21**	**21**	**19**	**19**	**10**	**9**	**7**
2	22	20	27	18	14	17	19
3	19	19	36	16	12	27	31
4	17	20	35	27	16	27	38
5	40	19	31	26	30	31	22
**Total**	**119**	**99**	**148**	**106**	**82**	**111**	**117**

*ASCOT-Carer Domains 1 = Occupation, 2 = Control over Daily Life, 3 = Self Care, 4 = Personal Safety, 5 = Social participation, 6 = Time and Space to be yourself, 7 = Feeling supported and encouraged

** CarerQol Domains 1 = Fulfilment, 2 = Relational Problems, 3 = Mental Health Problems, 4 = Problems combining daily activities with care, 5 = Financial problems, 6 = Support, 7 = Physical Health problems

***Carer Experience Scale Domains 1 = Activities outside Caring, 2 = Support from family and friends, 3 = Assistance from Organisations and Government, 4 = Fulfilment from Caring, 5 = Control over Caring, 6 = Getting on with the Care recipient

### Comprehensiveness

After completing the instrument, very few participants reported that the carer-related instruments covered all aspects affecting care-related QoL (ASCOT-Carer (n = 2), CarerQol (n = 3) and CES (n = 2). Content analysis of this question for each instrument (Online Appendix 2) shows that all themes in the coding framework were identified as missing by our sample of carers. This is a particularly interesting result, as many of the themes are constructs measured by the instruments.

## Discussion

This analysis identified aspects of carer QoL important to Australian informal carers and explored how applicable the constructs of ASCOT-Carer, CarerQol and CES were to this population. Behaviour-mood of care recipient, Caring Responsibilities, Finances, Health, Own Life, Perceptions of carers, Relationship with care recipient and Support were identified as aspects of caring that affect carer QoL in Australian.

Comparing domains most importance in our sample with previous studies that developed preference weights and tariffs for the instruments showed mixed results. Occupation and control over daily life for the ASCOT-Carer where the most preferred among English carers [21], whereas our Australian sample found self-care and time and space to be yourself to be the most important. Suggesting that, with further investigation, Australian preference weights for the ASCOT-Carer may be in need of development. In our sample the importance of domains in the CarerQol (most important, mental health; least important, combining care and other activities) and CES (most important, getting on with care recipient and activities outside of caring; least important, control over caring) were in line with instrument tariffs [7]. CarerQol tariffs, developed for Australia, indicated mental health and combining caregiving with other activities as the most and least preferred [22, 23]. Whilst in a sample from United Kingdom, the CES found activities outside of caring and getting on with the care recipient as most preferred and control over caring the least [7].

Almost all participants reported aspects of carer QoL not captured by the carer-related instruments. Many of these aspects that were perceived as not covered by the instruments, could have been included in the domains. This was also the case in a study looking at patient, self-reported, QoL aspects not captured by EQ-5D-5L [39]. As caring experiences are subjective and responsibilities and challenges vary greatly between carers, they may have felt the domain did not completely encompass their experience of carer QoL. This reflects how some aspects of QoL, which can be important to individuals, cannot necessarily be translated into a question for a QoL instrument. Particularly when required to be applicable to a broad range of carers (e.g. caring for partner, child, parent), align with the construct of the instrument, and also fit with other considerations (e.g., timeframe).

Alternatively, respondents may have interpreted the questions differently or focused only on certain portions of the question (e.g. heading, examples or explanatory text). A content analysis of the end-of-life patient-reported outcome measure showed that interpretation of questions is related to individual circumstances, where in financial matters varying themes of money, investments, funeral arrangements and wills emerged [40]. This could also explain why similar domains across instruments were treated differently. The domain of support is present in all three instruments, however, respondents reported different types of support were missing in each instrument (i.e. formal and informal support for the carer and/or care recipient). Comparable results were also found in more detailed studies of the exploratory factor analysis using this same dataset, where only a moderate correlation was found between CarerQol and CES support items and also for relational problems [17].

Content and/or face validation of the three instruments has not been performed with Australian carers, so detailed information of how each instrument's questions are interpreted and understood by this population is not known. The broader project performed a content comparison of the three instruments showing they each perform well in measuring their relevant domains with Australian carers [15, 17]. However, the qualitative component of this study suggests that some differences in question interpretation may exist.

The majority of sub-themes (over 50%) related specifically to the care recipient. However, only two domains include aspects of carer QoL that are influenced by the care recipient (CarerQol’s relational problems and CES’s getting on with the care recipient). Consideration was given to a similar domain in the development of the ASCOT-Carer, however, it was omitted because it did not fit with the construct of the instrument (social care-related QoL/impact of care services on carer QoL) [18].

This relationship between the care recipient and carer has previously been proposed as an advantage of the CES in capturing broader aspects of caring [7, 15, 16]. Given the possible interdependence of care recipient and carer QoL [41-43], instruments capturing both could be included in economic evaluations of carer and patient interventions to fully capture the effects of an intervention [44]. However, consideration also needs to be given to the type of evaluation being performed, the perspective taken and the possibility of double counting which could overestimate the benefits of an intervention [2, 45]. Keeping these factors and participant burden in mind, an appropriate combination of instruments may be used in measuring carer QoL in studies focused on informal carers.

Some of the missing aspects of carer QoL in the instruments, as reported by our sample, are intentionally not covered by the instruments as they each have been developed with different intentions and measure different constructs of CrQoL. The CarerQol was developed and intended to measure the impact/burden of caregiving on QoL and so, appropriately, does not include any themes specifically about the care recipient [6]. Similarly, the CES missing themes of finance and health are reasonably missing as the instrument’s purpose is to measure the experience of caregiving. Health problems are not directly measured by CES, as qualitative research indicated that this was linked to other attributes included in the instrument [19]. And the ASCOT-Carer does not measure finance and health, as the instrument was developed as a measure of social care and support services on carer QoL. Financial hardship due to caring and health were considered in the early development of ASCOT-Carer [31], however, it was excluded as it was outside the scope of the instrument’s purpose. Although health was not considered as a separate domain in the ASCOT-Carer it is captured by the lowest QoL (high-level needs) response option for each item and indicates that the carer has high-level needs that, if unmet over time, put the carer at risk of poor physical and/or mental health.

### Strengths and limitations

Content analyses have the potential to be influenced by researchers’ experiences and preconceptions. Coding framework along with transcripts were reviewed by a second researcher independently and collaboratively discussed, reducing the impact of coder bias. Quotes and sub-themes were classified to themes based on consensus and the coding framework is presented to demonstrate how the data were categorised so that other researchers can consider how their interpretation aligns with the researchers’ views.

The recruitment of study participants and completion of the questionnaires occurred towards the end of the roll-out of a new government support system, National Disability Insurance Scheme (NDIS), which replaced the existing system of disability support. The NDIS caused changes to administrative processes in receiving financial and formal support and may have been particularly front of mind for carers having to navigate this new system. Further, specific issues may have arisen directly due to changes in the systems.

The cohort included a greater number of sole carers (55 vs 33%), a greater proportion of female carers (79% vs 57%) and a higher percentage of carers providing more than 30 h of care per week (55% vs 45%) compared to the Australian population of primary carers [11, 12]. Income and employment, relationship to care recipients and sharing household with care recipient were similar to the Australian population of carers [12]. Uniquely, participants included carers of people with multiple health conditions. The study included informal carers in the Australian setting only and therefore results may have limited generalisability to other settings.

Study participants were self-selected via newsletter advertising and, to reduce burden, randomly allocated to one additional activity described in this paper (excluding the ranking activity that was completed by all). This resulted in a different sub-group completing each (Positive/Negative question, the three [Instrument] missing domain questions, the three [Instrument] domain importance questions). Sub-group characteristic differences (Online Appendix 1) include the Positive/Negative question participants containing only females, being less employed, less likely to be married and more likely to have been caring for > 24 months and > 4 h of care per week and the CES missing domain participants being less educated and caring for more recipients with mental health problems.

The open-ended components of this study were embedded in a larger quantitative study [15, 17], so there was no opportunity to apply qualitative techniques such as face-to-face interviews or focus groups to explore responses in more depth, clarify the views’ expressed or to measure comprehensibility of the instrument questions. Cognitive interviewing, in checking respondent’s understanding, mentally processing and response to materials would help with understanding these differences. Cognitive interviewing evidence is present for the ASCOT-Carer in England [18, 31, 32] and during its translation into German [46, 47], Japanese [48] and Finnish [49], as well as for the CES in England [19]. Australian evidence would provide a much greater understanding of the comprehensiveness and comprehensibility of the three instruments in this setting.

As questionnaires were completed anonymously, researchers did not have an avenue to discuss findings with participants and receive feedback on the themes and analysis.

Importance of domains in this study, were assessed by a sample of informal carers. Whereas carers also participated in the development of preference weights for the CES using a best-worse scale (BWS) valuation exercise [7], the ASCOT-Carer and CarerQol used the general population imagining a hypothetical state of being an informal carer using BWS exercise and a discrete choice experiment respectively [21, 22]. The inconsistencies with our sample may be due to the differences in sample (i.e., carers or general population) and also between stated different methodology using preferences (hypothetical situation) or revealed preferences (actual or current situation) or due to different analyses.

Three different approaches were used to investigate how well the instruments capture aspects of CrQoL important to Australian informal carers, strengthening conclusions concerning coverage.

Some potential cultural/ethnic difference appear in the free text responses. However, with a very small number of participants born outside of Australia and/or speaking a language other than English (Online Appendix 1) these differences were not explored in this paper. A real opportunity exists for future research in this area of an Australian population.

## Conclusions

Open-ended responses and quantitative data collected from a sample of Australian informal carers, suggest there are multiple aspects of caring that impact carer QoL. Consideration should be given to measurement of care recipient health and well-being and spillover effects affecting carer QoL, with thought to the risk of double counting.

The ASCOT-Carer, CarerQol and CES appear to be relevant for an Australian informal carer population and include most of the aspects of quality of life important to them. The interpretation of questions may differ in Australian informal carers which requires confirmation with a content and/or face validity assessment.

The findings support previous research that the selection of an instrument should take into account the aim, purpose and constructs of the instrument.

### Supplementary Information

Below is the link to the electronic supplementary material.

Supplementary file1 (DOCX 23 kb)
