# Supplementary material for: How well do the adult social care outcomes toolkit for carers, carer experience scale and care-related quality of life capture aspects of quality of life important to informal carers in Australia?
Source: Qual Life Res. 2023 Jun 25;32(11):3109–21. doi: 10.1007/s11136-023-03459-1 (PMC10522516; doi:10.1007/s11136-023-03459-1)
Supplement: Supplementary file 1 — Supplementary file1 (DOCX 23 kb) [file 11136_2023_3459_MOESM1_ESM.docx]

# Appendix 1 – Sample characteristics by task

| **Characteristic** | **Total sample** | **Positive/ Negative**  **question** | **[Instrument]**  **Carer rating of missing domains question*** | | | **[Instrument]**  **Carer rating of domain importance question*** | | | **Ranking Activity** |
| --- | --- | --- | --- | --- | --- | --- | --- | --- | --- |
|  |  |  | **ASCOT-Carer** | **CarerQol** | **CES** | **ASCOT-Carer** | **CarerQol** | **CES** |  |
| **Sample size (completed questions)** | **500** | **28** | **24** | **33** | **30** | **103** | **72** | **58** | **449** |
| **Informal Carer** |  |  |  |  |  |  |  |  |  |
| Age in years: mean (SD) | 52 (14) | 58 (10) | 55 (12) | 53 (12) | 53 (14) | 56 (14) | 50 (16) | 51 (14) | 52 (14) |
| Gender, female (n=497): n (%) | 393 (79) | 28 (100) | 20 (83) | 29 (88) | 24 (80) | 82 (80) | 50 (69) | 47 (81) | 349 (78) |
| Education attainment: n (%) |  |  |  |  |  |  |  |  |  |
| Year 11 or below | 87 (17) | 3 (11) | 4 (17) | 2 (6) | 8 (27) | 18 (17) | 13 (18) | 8 (14) | 77 (17) |
| Year 12 | 44 (9) | 2 (7) | 3 (13) | 2 (6) | 4 (13) | 7 (7) | 1 (1) | 4 (7) | 37 (8) |
| Certificate/ Diploma | 137 (27) | 8 (29) | 4 (17) | 10 (30) | 10 (33) | 27 (26) | 19 (26) | 18 (31) | 125 (28) |
| Undergraduate | 131 (26) | 7 (25) | 9 (38) | 10 (30) | 4 (13) | 26 (25) | 23 (32) | 19 (33) | 118 (26) |
| Postgraduate | 90 (18) | 8 (29) | 4 (17) | 9 (28) | 4 (13) | 22 (21) | 15 (21) | 8 (14) | 82 (18) |
| Other | 7 (1) | 0 (0) | 0 (0) | 0 (0) | 0 (0) | 3 (3) | 1 (1) | 1 (2) | 7 (2) |
| Employment: n (%) |  |  |  |  |  |  |  |  |  |
| Employed (full-time, part-time, casual, self-employed) | 253 (51) | 6 (21) | 9 (38) | 11 (33) | 11 (37) | 53 (51) | 38 (53) | 35 (60) | 221 (49) |
| Retired, housework duties including carer tasks | 229 (46) | 21 (75) | 15 (63) | 19 (58) | 17 (57) | 49 (48) | 30 (42) | 23 (40) | 212 (47) |
| Student, unemployed or other | 16 (3) | 1 (4) | 0 (0) | 3 (9) | 2 (7) | 1 (1) | 3 (4) | 0 (0) | 15 (3) |
| Country of birth, Australia: n (%) | 401 (80) | 21 (75) | 19 (79) | 22 (67) | 25 (83) | 81 (79) | 63 (88) | 48 (83) | 364 (81) |
| Language spoken at home, English (n=499): n (%) | 478 (96) | 26 (93) | 23 (96) | 30 (91) | 27 (90) | 98 (96) | 71 (99) | 55 (97) | 431 (96) |
| Marital status: n (%) |  |  |  |  |  |  |  |  |  |
| Single | 39 (8) | 2 (7) | 2 (8) | 2 (6) | 2 (6) | 3 (3) | 5 (7) | 2 (3) | 37 (8) |
| Married or de-facto | 371 (75) | 16 (57) | 18 (75) | 25 (76) | 25 (76) | 82 (80) | 53 (74) | 47 (81) | 336 (75) |
| Divorced, separated or widowed | 83 (17) | 9 (32) | 4 (17) | 5 (15) | 5 (15) | 17 (17) | 13 (18) | 9 (16) | 70 (16) |
| Annual household income: n (%) |  |  |  |  |  |  |  |  |  |
| <$52,000 | 203 (47) | 14 (50) | 14 (58) | 13 (39) | 13 (39) | 35 (34) | 29 (40) | 21 (36) | 184 (41) |
| $52,000 - $129,999 | 175 (41) | 5 (18) | 5 (21) | 13 (39) | 13 (39) | 43 (42) | 23 (32) | 16 (28) | 161 (36) |
| $130,000 or more | 50 (12) | 1 (4) | 1 (4) | 2 (6) | 2 (6) | 9 (8) | 12 (17) | 12 (21) | 42 (9) |
| **Care recipient** |  |  |  |  |  |  |  |  |  |
| Age: mean (SD) | 45 (26) | 48 (29) | 57 (24) | 51 (31) | 41 (27) | 49 (28) | 45 (23) | 48 (25) | 45 (26) |
| Gender, female: n (%) | 202 (40) | 12 (43) | 8 (33) | 18 (55) | 11 (37) | 39 (38) | 33 (46) | 20 (34) | 186 (42) |
| Medical condition (multiple answers): n (%) |  |  |  |  |  |  |  |  |  |
| Temporary disease or disability | 34 (7) | 1 (4) | 3 (13) | 2 (6) | 1 (3) | 5 (5) | 9 (13) | 5 (9) | 33 (7) |
| Chronic disease or disability | 218 (44) | 13 (46) | 11 (46) | 12 (36) | 12 (40) | 50 (49) | 33 (46) | 20 (35) | 197 (44) |
| Dementia or memory problems | 110 (22) | 9 (32) | 7 (29) | 10 (30) | 3 (10) | 23 (22) | 16 (22) | 13 (22) | 100 (22) |
| Mental health problems | 164 (33) | 10 (36) | 9 (38) | 7 (21) | 18 (60) | 27 (26) | 25 (35) | 17 (29) | 146 (33) |
| Intellectual or developmental disability | 164 (33) | 8 (29) | 8 (33) | 12 (36) | 14 (47) | 33 (32) | 16 (22) | 11 (19) | 146 (33) |
| Problems due to aging | 88 (18) | 8 (29) | 6 (25) | 11 (33) | 5 (17) | 28 (27) | 10 (14) | 8 (14) | 81 (18) |
| Terminal illness | 25 (5) | 0 | 2 (8) | 1 (3) | 0 (0) | 6 (6) | 4 (6) | 3 (5) | 25 (6) |
| Neurological | 16 (3) | 0 | 2 (8) | 0 (0) | 1 (3) | 5 (5) | 4 (6) | 0 (0) | 15 (3) |
| Other | 7 (1) | 1 (4) | 1 (4) | 0 (0) | 0 (0) | 3 (3) | 0 (0) | 1 (2) | 7 (2) |
| Number of medical conditions: mean (SD) | 1.74 (0.93) | 1.79 (0.99) | 2.12 (1.26) | 1.67 (0.81) | 1.97 (0.93) | 1.8 (0.98) | 1.63 (0.99) | 1.40 (0.62) | 1.75 (0.94) |
| **Caring situation** |  |  |  |  |  |  |  |  |  |
| Relationship to the care recipient: n (%) |  |  |  |  |  |  |  |  |  |
| Partner | 126 (25) | 6 (21) | 9 (38) | 6 (18) | 4 (13) | 26 (25) | 21 (29) | 15 (26) | 120 (27) |
| Child (Daughter/son) | 161 (32) | 14 (25) | 5 (21) | 11 (33) | 14 (47) | 36 (35) | 19 (26) | 15 (26) | 139 (31) |
| Parent (mother/father) | 157 (32) | 7 (25) | 7 (29) | 12 (36) | 9 (30) | 29 (28) | 24 (33) | 25 (43) | 139 (31) |
| Another family member, friend or neighbour | 51 (10) | 0 | 2 (8) | 4 (12) | 3 (10) | 11 (11) | 7 (10) | 3 (5) | 47 (10) |
| Sole carer, yes: n (%) | 269 (55) | 20 (71) | 13 (54) | 20 (60) | 15 (50) | 56 (54) | 45 (63) | 31 (53) | 239 (53) |
| Support more than one care recipient, yes: n (%) | 129 (26) | 5 (29) | 6 (25) | 6 (18) | 12 (40) | 29 (28) | 20 (28) | 15 (26) | 108 (24) |
| Sharing household with care recipient, yes: n (%) | 402 (81) | 22 (79) | 19 (79) | 24 (72) | 27 (90) | 84 (82) | 53 (74) | 44 (76) | 367 (82) |
| Duration of care: n (%) |  |  |  |  |  |  |  |  |  |
| ≤24 months | 128 (26) | 1 (4) | 3 (13) | 4 (12) | 4 (13) | 26 (25) | 21 (29) | 25 (43) | 115 (26) |
| >24 months | 370 (74) | 27 (96) | 20 (83) | 29 (88) | 26 (87) | 77 (75) | 51 (71) | 33 (57) | 332 (74) |
| Hours of care per week: n (%) |  |  |  |  |  |  |  |  |  |
| <20 hours | 100 (20) | 3 (11) | 5 (21) | 8 (24) | 2 (7) | 28 (27) | 17 (24) | 16 (28) | 88 (20) |
| 20 – 29 hours | 112 (23) | 3 (11) | 5 (21) | 6 (18) | 7 (23) | 19 (18) | 16 (22) | 17 (29) | 98 (22) |
| 30 – 39 hours | 59 (12) | 4 (14) | 0 (0) | 3 (9) | 6 (20) | 8 (8) | 9 (13) | 8 (14) | 56 (13) |
| ≥40 hours | 217 (43) | 17 (61) | 12 (50) | 15 (45) | 15 (50) | 47 (46) | 29 (40) | 17 (29) | 199 (44) |

*No duplicate participants between any [Instrument] missing domain questions and [Instrument] domain important questions. Each of these six sub-groups are independent.

# Appendix 2 - Content Analysis Coding Framework

* Shaded cells indicate that the sub-theme (row) was present in responses to specific question (column).

|  | |  | **[Instrument] Missing domain questions** | | | **Positive/Negative questions** | |
| --- | --- | --- | --- | --- | --- | --- | --- |
| **Theme** | **Sub-theme 1** | **Sub-theme 2** | **ASCOT- Carer** | **CarerQol** | **CES** | **Neg** | **Pos** |
| **Behaviour-Mood of care recipient** | Abuse |  |  |  |  |  |  |
|  | Accomplishment |  |  |  |  |  |  |
|  | Comfort |  |  |  |  |  |  |
|  | Cooperation |  |  |  |  |  |  |
|  | Happiness |  |  |  |  |  |  |
|  | Suspicious |  |  |  |  |  |  |
|  | Unpredictability |  |  |  |  |  |  |
|  | Unspecified |  |  |  |  |  |  |
|  | Upset |  |  |  |  |  |  |
| **Caring Responsibilities** | Advocating |  |  |  |  |  |  |
|  | Reason for Caring |  |  |  |  |  |  |
|  | Travel |  |  |  |  |  |  |
| **Finances** | Costs |  |  |  |  |  |  |
|  | Income-Employment |  |  |  |  |  |  |
| **Health** | of care recipient | Mental |  |  |  |  |  |
|  |  | Physical |  |  |  |  |  |
|  | of carer | Mental |  |  |  |  |  |
|  |  | Physical |  |  |  |  |  |
|  |  | Spiritual |  |  |  |  |  |
|  |  | Fatigue |  |  |  |  |  |
| **Own Life** | Caring-Life balance |  |  |  |  |  |  |
|  | Exercise |  |  |  |  |  |  |
|  | Family |  |  |  |  |  |  |
| **Perceptions of carers** |  |  |  |  |  |  |  |
| **Relationship with care recipient** | Change |  |  |  |  |  |  |
|  | Communication |  |  |  |  |  |  |
|  | Feelings toward |  |  |  |  |  |  |
|  | Time together |  |  |  |  |  |  |
| **Support** | for care recipient | Formal |  |  |  |  |  |
|  |  | Informal |  |  |  |  |  |
|  | for carer | Formal |  |  |  |  |  |
|  |  | Informal |  |  |  |  |  |
